# Review of recent developments in iodine wasteform production

**DOI:** 10.3389/fchem.2022.1043653

**Published:** 2022-12-23

**Authors:** R. Matthew Asmussen, Joshua Turner, Saehwa Chong, Brian J. Riley

**Affiliations:** ^1^ Pacific Northwest National Laboratory, Richland, WA, United States; ^2^ National Nuclear Laboratory, Sellafield, Cumbria, United Kingdom

**Keywords:** iodine, nuclear reprocessing, wasteforms, I-129, disposal

## Abstract

Radioiodine capture and immobilization is not only important to consider during the operation of reactors (i.e., I-131), during nuclear accidents (i.e., I-131 and I-129) or nuclear fuel reprocessing (i.e., I-131 and I-129), but also during disposal of nuclear wastes (i.e., I-129). Most disposal plans for I-129-containing waste forms (including spent nuclear fuel) propose to store them in underground repositories. Here, iodine can be highly mobile and, given its radiotoxicity, needs to be carefully managed to minimize long-term environmental impacts arising from disposal. Typically, any process that has been used to capture iodine from reprocessing or in a reactor is not suitable for direct disposal, rather conversion into a wasteform for disposal is required. The objectives of these materials are to use either chemical immobilization or physical encapsulation to reduce the leaching of iodine by groundwaters. Some of the more recent ideas have been to design capture materials that better align with disposal concepts, making the industrial processing requirements easier. Research on iodine capture materials and wasteforms has been extensive. This review will act as both an update on the state of the research since the last time it was comprehensively summarized, and an evaluation of the industrial techniques required to create the proposed iodine wasteforms in terms of resulting material chemistry and applicability.

## Graphical Abstract

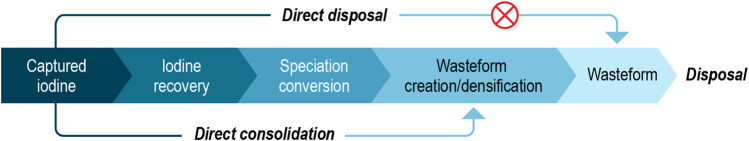

## 1 Introduction

Nuclear fission is a dense source of energy that produces fission products. Radioiodine primarily includes two fission products that attract attention due to the potential for biological uptake if released into the environment, which are short-lived I-131 (*t*_1/2_ = 8.04 d) and the longer-lived I-129 (*t*_1/2_ = 1.57 × 10^7^ y). The full life cycle of iodine is important to understand including within the fuel (Saidy et al., 2008; Konings et al., 2015; Heikinheimo et al., 2021), its release during fuel reprocessing (Soelberg et al., 2013), its release during disposal (Schreinemachers et al., 2022), technologies that can be used to abate the released iodine (Krumhansl et al., 2006; Riley et al., 2016), and methods of immobilizing iodine for long-term disposal and iodine behavior in the environment (Moran et al., 2002; Santschi et al., 2017; Neeway et al., 2019; Moore et al., 2020). In this review, the focus is placed on methods of immobilizing iodine into wasteforms suitable for long-term disposal by updating the review on the capture of iodine (Riley et al., 2016) and filling a crucial hole in the literature for a comprehensive review of the conversion of radioiodine-laden materials to wasteforms.

The interest around the long-term management of iodine is accentuated because iodine is readily up taken into the biosphere and humans, specifically concentrating in the thyroid (Drozdovitch, 2021). For reactor operations, I-131 is the isotope of concern due to its short *t*_1/2_ but high activity, whereas in reprocessing and disposal, I-129 becomes important due to the long timeframes considered. Whilst the individual dose from I-129 is significantly smaller than I-131, the effect of aggregating a large volume of used nuclear fuel or iodine-containing wasteforms in a repository could create a potentially large and sustained source term.

Radioiodine is generated during fission and does not incorporate well into the UO_2_ fuel matrix (Konings et al., 2015). As a result, iodine is quite mobile in the fuel and can be found in the headspace of the fuel assembly. If a used fuel container within a repository is breached by infiltrating water, the iodine within the headspace is available for immediate release, which is followed by a slower release of iodine from within the fuel pellet. If the fuel is reprocessed prior to disposal, the fate of iodine differs.

**FIGURE 1:**
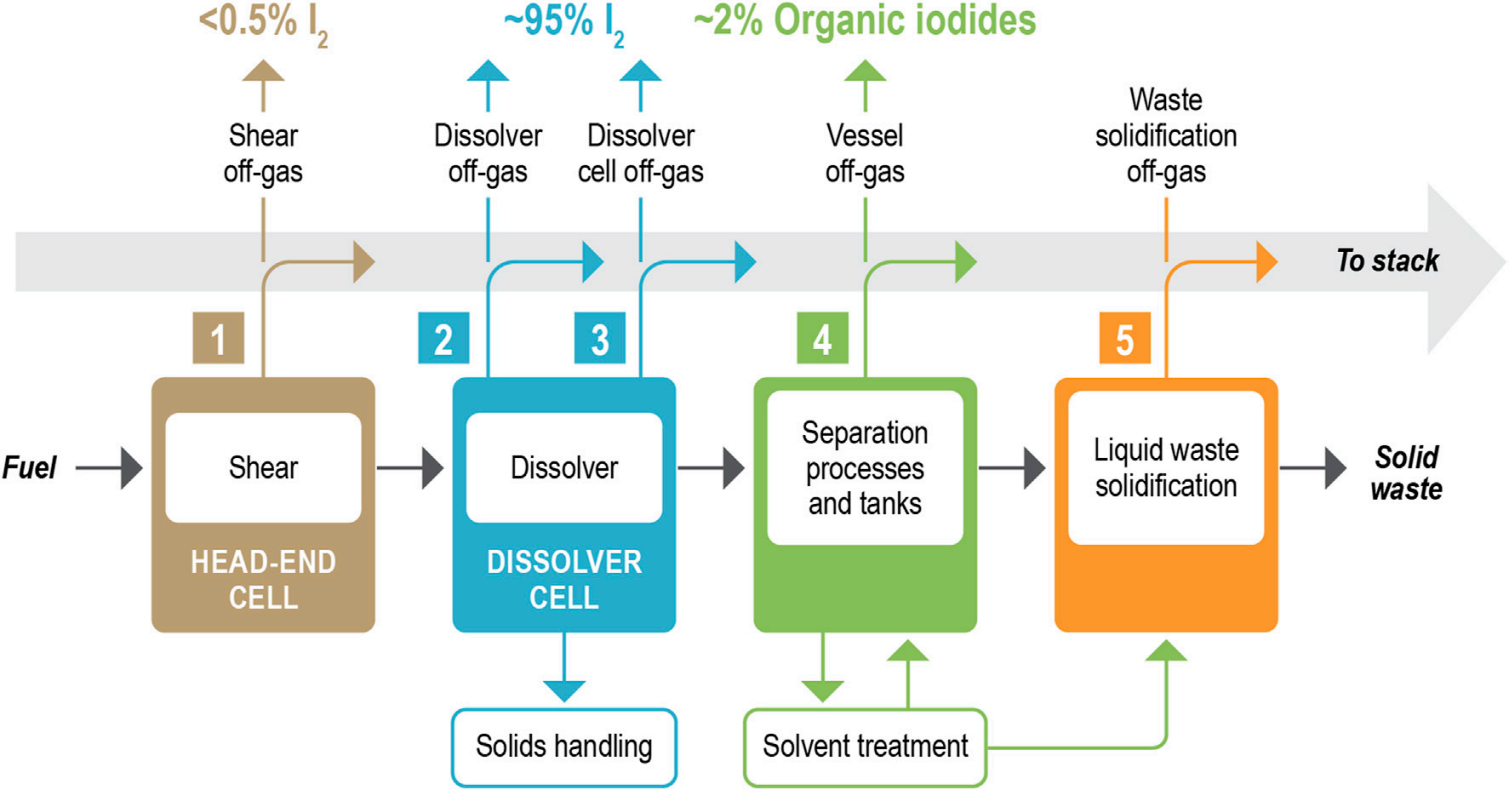
Schematic of iodine partitioning during aqueous reprocessing of used nuclear fuel.

When nuclear fuel is reprocessed, it is first dissolved in acid (commonly in HNO_3_) where the majority of iodine (94%–99%) is released to the dissolver off-gas system (Figure 1) (Sakurai et al., 1992). Some of the iodine forms part of the insoluble fission products [e.g., PdI_2_ (Buck et al., 2016)], whilst the remainder persists downstream. Once in the solvent separation plant, the remaining iodine can be released as organic iodide (e.g., CH_3_I, C_2_H_5_I). The majority of released iodine is targeted to be captured in the off-gas management train, historically using a liquid scrubbing process (Hudson and Buckley, 1996; Treatment of Radioactive Gaseous Waste, 2014) or Ag-based sorbent (Soelberg et al., 2013). Liquid scrubbing processes include caustic scrubbing, the Mercurex process, and the Iodox process that result in spent liquors that are challenging to treat and instead use a liquid discharge, which is of lower consequence than aerial discharge. For short-cooled high-burnup fuel, the Mercurex process has been used on plants such as the Prototype Fast Reactor (PFR) reprocessing plant to achieve the required degree of removal (Hebel and Cottone, 1982). For solid sorbents, these are typically porous scaffolds like molecular sieves (e.g., zeolite A, faujasite, mordenite) (Maeck et al., 1970; Pence et al., 1970; Riley et al., 2022), or emerging materials like aerogels (Matyas et al., 2018), or xerogels (Chong et al., 2021; Kang et al., 2022) with active getter sites (e.g., Ag^+^, Ag^0^) designed to scavenge iodine to form metal-iodide complexations through a chemisorption process (Treatment of Radioactive Gaseous Waste, 2014). In France at La-Hague these solid sorbents are deployed to increase the decontamination factors (DFs) (Parisot, 2008). At the Tokai Mura plant in Japan, a silver faujasite has been used (Treatment of Radioactive Gaseous Waste, 2014). Silver saddles have been used during reprocessing at the Hanford site (Raab and Van der Cook, 1970). While Ag is the most commonly investigated element for iodine capture other elements also form low soluble complexes with iodide or iodate, such as Pb (Clark, 1977), Bi (Levitskaia et al., 2022) and lesser tested elements like Gd (Jing-Jing et al., 2020).

Substantial efforts have been placed at developing materials for the abatement of iodine from reprocessing off-gases with improved efficiency, stability, and economics albeit with low technology readiness levels, which is a goal for many other radionuclides (Jun-Hao et al., 2020). These iodine removal efforts have included the use of metal loaded zeolites (Jabraoui, 2019; Al-Mamoori et al., 2020), layered double hydroxides (Dinh et al., 2020), porous silica/alumina materials (Yang et al., 2015; Jubin et al., 2018), macroreticular resins (Daryl Haefner, 2007), aerogels/chalcogels/xerogels (Riley et al., 2020), activated carbons (Sachin U. Nandanwar S. U. et al., 2016), titania/titaniasilocates (Wu et al., 2014), porous organic polymers (Wang et al., 2020), and metal-organic frameworks (Han et al., 2020; Zhang et al., 2022). For good reviews of iodine capture methods, see previous literature on the gaseous phase (Huve et al., 2018; S.U. Nandanwar S. U. et al., 2016; Pénélope et al., 2022a; Riley et al., 2016; Treatment of Radioactive Gaseous Waste, 2014; Xie et al., 2019) and from the aqueous phase (Robshaw et al., 2021).

Once the iodine has been captured, the materials will typically require further treatment if the goal is to dispose the materials using a repository concept. This conversion from capture material to wasteform is critical to ensure adequate disposal performance properties are met, but the conversion process must also be economically and technically feasible. However, this conversion and disposal approach has not always been the case. For large reprocessing plants such as Thorp and La Hague, disposal involves discharge of the iodine into a large body of water diluting the dose with natural iodine (I-127) (Treatment of Radioactive Gaseous Waste, 2014). Other processes involve capturing iodine on solid sorbents followed by interim storage. The reason for post-processing is the properties that enable efficient capture, such as open porosity, are not favorable for long-term stability. To understand the desirable properties of the wasteform it is useful to consider various disposal scenarios.

One of the most favorable long-term solutions to the disposal of spent nuclear fuel and other higher activity wastes is a deep geological disposal facility (Kurniawan et al., 2022). Many countries across the world are still developing their concepts, which means that the environments that wasteforms are expected to experience are uncertain. This provides a compelling reason to ensure that sufficient wasteform diversity exists to deal with specific environmental factors unique to different disposal sites. The most relevant variable related to iodine wasteform durability is the redox conditions of the site. In an oxidizing environment (e.g., shallow subsurface), AgI is stable and the preferred Ag state in the wasteform. In this review, AgI is referred to as stabilized iodine. Whereas in reducing conditions, AgI can be displaced/reduced and an alternate form, such as NaI, may be prevalent. Here, NaI and NaIO_3_ (or other water-soluble salts) are referred to as soluble iodine in this review.

For the geological disposal concept, iodine can provide a significant contribution to the radiation dose experienced on the surface if I-129 is released to the environment. The factors that determine the overall dose from the wasteform depend on the immediate wasteform durability upon contact with infiltrating water, repository containment, and the surrounding geology and hydrology. Salt-based rock geologies, for example, hinder migration of iodine to the surface, whereas granite leads to facile mobility due to the much shorter transport times through the geosphere (Altmann, 2008; Grambow, 2008; Vaughn et al., 2012). Whilst retaining iodine in the wasteform on timescales comparable to I-129 decay is challenging, reducing the peak dose experienced by the surface is potentially easier. If the iodine release from the wasteform can be slowed, the iodine will reach the surface over a longer time period. This approach would provide the opportunity for the iodine to dilute and disperse within the biosphere.

Clearly, the choice of wasteform, and associated conversion process, can have an important role in mitigating the migration of iodine to the surface from a repository. Wasteforms that retain iodine have been under investigation for decades, but so far none have seen large-scale deployment. An optimization problem exists for meeting the technical needs of minimizing dose once disposed, and the economic needs of ensuring that a closed fuel cycle is competitive. The conversion from capture material to wasteform is central to mitigation of this problem. There have been a number of reviews of various options for iodine wasteforms that provide a good basis for the field (Burger et al., 1981; Hebel and Cottone, 1982; Trevorrow et al., 1983; IAEA, 1987; Taylor, 1990; Ojovan et al., 2019). This work aims to provide a comprehensive update on reports of iodine wasteforms over the last 6 years (Riley et al., 2016) and expanding on the wasteform types reviewed previously. It augments the work from a 2022 paper (Pénélope et al., 2022a), which contained an excellent summary of wasteforms that have been developed to target specific capture concepts. This review will place emphasis on the critical component of consolidation technologies used, as these will have a key role in determining the practicalities of deploying a wasteform concept at an industrial scale and ensuring disposal performance.

## 2 Industrial processing techniques for iodine consolidation

The eventual processing of particulate iodine-loaded materials to wasteforms will need to be done at a large scale with any technology that is selected previously demonstrated at scale. As such, having prior industrial application of the technology in a range of fields will provide defense-in-depth for any technology down-selection. Several relevant processes to eventual wasteform production and industrial experience related to iodine processing are present in the literature.

### 2.1 Hot pressing

Hot pressing is used industrially for the densification and removal of pores from materials under applied temperature. The conventional approach is uniaxial/unidirectional pressing in which the material to be densified is placed in a die and a punch is used to compress the encased material under heating. The continued contact between the punch and the material leads to compression with unevenly distributed friction and force (Atkinson and Davies, 2000), leading to heterogenous densification. Heterogeneity is not a desirable wasteform trait due to the likelihood of incongruent dissolution, uneven porosity or uneven distribution of contaminants.

These concerns can be removed by using hot isostatic pressing (HIP) where the material is placed within a hermetically sealed container made of a metal that can undergo plastic deformation at the pressure and temperature used during the process. As pressure is applied and the container is heated, the material will begin to densify and the walls will shrink somewhat uniformly from the isostatic pressure. The pressing can lead to a near complete removal of voids within the material (Atkinson and Davies, 2000). HIPing has been applied industrially for decades and to a range of materials including aluminum alloys (Ceschini et al., 2008), titanium alloys (Xu et al., 2018), copper alloys, ceramics (Bocanegra-Bernal, 2004; Thornber et al., 2018), steel (Antona and Mapelli, 2001), and apatites (Adolfsson et al., 2000). The original development of HIP was to produce nuclear components through diffusion bonding in the 1960s. HIP was considered for waste immobilization in the 1970s (Larker, 1979) and has been pursued further for the densification of radioactive materials including Kr-85 canisters (Pinchback, 1979), ceramic wastes (Bateman et al., 2002), mordenite (above) (Bruffey et al., 2015), and most recently for materials related to the Fukushima-Daiichi clean up mission (Gardner et al., 2021). Due to the hermetic sealing, HIP is more desirable than uniaxial pressing as the loss of iodine from volatilization during HIP is non-existent without container breach. The removal of void space can also serve to protect a large amount of the contaminant inventory from exposure to the environment upon disposal. The industrial experience with HIP is promising, however handling radioactive materials under high temperatures and pressures will require significant safety controls.

### 2.2 Sintering

Sintering is a process through which a powder is transformed into solid or porous mass by heating and avoiding liquefaction. Sintering has long been used in the fabrication of nuclear fuel converting UO_2_ powders to a densified pellet (Greenquist et al., 2018). Traditional sintering can take hours to perform, however the process can be accelerated through several means. Spark plasma sintering (SPS), which is also known as pulsed electric current sintering (PECS) and field-assisted sintering technique (FAST), uses a pulsed direct current voltage along with uniaxial force under controlled pressure to densify a powder in a graphite die. As the current is pulsed, sparks are generated creating local high temperatures inducing a connection coalescence between the particles (Ogunbiyi et al., 2019). The material is heated (upwards of 2000°C) with very high heating rates directly if it is conductive while non-conductive materials can be heated by employing a conductive punch and die. This variety allows different material types to be processed with SPS (e.g., metals, ceramics and polymers) (Guillon et al., 2014).

Traditional sintering could lead to significant volatilization of iodine in processing of a wasteform due to the long process times required. This risk would be reduced in the use of SPS due to the shorter densification times on the order of minutes, dependent on processing conditions. However, the SPS process is far less technically mature but seeing growing use in industry. The risks associated with high temperatures, pressure, and use of electrical currents requires proper safety control for any expanded use in waste immobilization.

Microwave sintering could also be used in which the heat source for the sintering process comes from the adsorption of microwaves (Rybakov et al., 2013). The main advantages of microwave sintering are the high volumetric heating adsorption, efficiency as the input power is nearly entirely used on microwave generation, and the production of finer materials with fewer defects (Borrell, 2018). In the nuclear field microwave sintering has been explored for the immobilization of contaminated soils (S. Zhang et al., 2017b), production of garnet wasteforms (Zhang et al., 2021), and geopolymer conversion (Xiang et al., 2021), but these are still developmental.

### 2.3 Vitrification

Vitrification has been used for the immobilization of nuclear wastes on a large scale (Vienna, 2010) and most extensively in France (Vernaz and Bruezière, 2014), United Kingdom (Harrison, 2014), and the United States (Goel et al., 2019). The waste (i.e., liquid, calcined or particulate) is mixed with glass-forming chemicals, heated, and, upon cooling, produces a glass. Five types of technologies can be used including Joule heated ceramic melters, hot crucible induction melters, cold crucible melters, indirect heating, and in-can melters; their industrial uses are summarized elsewhere (McCloy and Schuller, 2022). Vitrification can generate durable amorphous materials that incorporate a wide range of elements to target specific properties or immobilization of complex species. The challenges of vitrification arise from the handling of the melter off-gas, which will contain volatile elements. Included in the volatile species is iodine, which makes vitrification challenging for the production of an iodine wasteform. Low-temperature vitrification processes are being developed, which could overcome these challenges (Ojovan et al., 2021).

Vitrification can also be performed on a small scale using in-can melters, also referred to as in-container vitrification, where waste is vitrified through a Joule heated process in a refractory container. The container would also serve as the disposal canister for the wasteform. In-can melting has been used in several efforts, mainly using the GeoMelt^®^ system, for chlorinated organics (Thompson, 2002), reactive metals (Campbell et al., 2018), depleted uranium (Finucane and Campbell, 2006), asbestos wastes (Finucane et al., 2008), and for use in clean-up at Fukushima (Didierlaurent et al., 2019). With the smaller volumes of iodine capture materials compared with liquid waste campaigns, in-can melting could be considered a deployable technology, as long as iodine retention can be ensured.

### 2.4 Ambient temperature processing

Cementitious and other low-temperature stabilization materials are a widely used technology in the nuclear industry for the stabilization of debris wastes due to their low cost, process simplicity, low risk of volatilization of species like iodine, and previous history of implementation (Bart et al., 2012; Scheele and Wend, 2015; Abdel Rahman and Ojovan, 2021). Converting iodine-loaded materials, such as silver mordenite (AgZ), to a final wasteform through stabilization in cementitious materials has long been studied and considered a viable approach for low-level wastes. In general, these processes involve a hydration reaction between dry reagents and water, generating a combination of crystalline and amorphous hydrated products that generate a solid mass close to ambient temperatures. The water can be a liquid waste containing iodine (e.g., I^−^, IO_3_^−^) or the resulting slurry can be mixed with particulate iodine-loaded materials. While the resulting wasteforms have porosity, they can be engineered to have desirable chemical properties to support wasteform deployment. In fact, these technologies are some of the oldest known to civilization (Jackson et al., 2009). Limitations may be realized depending on regulatory disposal requirements.

### 2.5 Consolidation summary

In summary there are several strong, industrially mature or emerging approaches that can be used for the conversion of iodine-loaded capture materials into final wasteforms. Technologies with high maturity are likely easier to transfer to radiological applications and should be considered in any further development of iodine wasteforms.

## 3 Iodine wasteforms

The development of an iodine wasteform is interdependent with the method of capturing it, where the choices made for capture can impact the required steps to reach a waste form. The more steps required to reach a final wasteform, the more complex the industrial process would have to be. A wasteform that requires the fewest steps from iodine capture to the resulting wasteform may have desirable characteristics (e.g., lower cost) relative to a complex but more durable wasteform. A recent review from this production perspective made direct connections between the common capture materials (e.g., mordenite) under consideration and select wasteforms (Pénélope et al., 2022a), although the list was not exhaustive. This current review expands on that work to also include wasteforms where a link to a capture material is unclear.

Following sequestering iodine from a waste stream, stabilized iodine (e.g., AgI) or soluble/sorbed iodine (e.g., NaI, NaIO_3_) are possible product forms that will be covered in this review; with each carrying their own benefits. The main drawbacks for keeping iodine bound in an Ag-based matrix is cost (without recycling) and toxicity. The trade-off is that AgI is one of the most chemically durable states of iodine in iodine-based wasteforms exhibiting very low leach rates (Reiser et al., 2022). However, in reducing environments, the Ag can be reduced to Ag^0^, leading to iodine release into solution.

Conversion of AgI to soluble NaI is possible. Feedstocks of AgI can be generated in large quantities during iodine capture by direct reaction of Ag metal with I_2g)_ (Jun-Hao et al., 2020). If desired, the iodine may be removed from AgI and converted into soluble I^−^ through a reaction with Na_2_S forming insoluble Ag_2_S that could be recycled into future sorbents (Cao et al., 2017). This NaI solution could then be immobilized in a separate wasteform such as an iodosodalite produced using aqueous methods (Chong et al., 2018; Riley et al., 2021).

The work by Riley and co-workers categorized different iodine wasteforms which are discussed in this section (Riley et al., 2016). Updates have been provided for new reports since that review, but the following categories of wasteform are not discussed: cancrinite, titanate ceramics, bismuth oxides, iodoboracite, silicon carbide, and direct disposal of iodide or iodate salts.

### 3.1 Vitrified wasteforms

A vitrified iodine wasteform can be produced from feeding a liquid waste or a loaded capture material to a melter. The incorporation of iodine into glass wasteforms is perceived to be challenging due to the volatility of iodine leading to low retention. Assessing iodine retention in glass materials is also challenging due to limited datasets and historical detection challenges of iodine in the solid (Langowski et al., 1996; Choi, 2020).

#### 3.1.1 Borosilicate glass

As stabilized or soluble iodine is introduced to a high temperature melt (>1000°C), a common conservative assumption is that both will behave similarly and evolve to the off-gas. Iodine is regularly observed to evolve from melter tests at >80% the feed inventory (DF of 1.25) regardless of melter type tested (Nakaoka et al., 1996; Shade, 1996; Gin et al., 2017). As such, development has focused heavily on improving iodine retention when vitrifying to convert to a borosilicate glass wasteform.

Previously iodine was thought to have low solubility within borosilicate glass; however, the retention is driven more by volatility than solubility. This observation has been made due to testing in sealed ampoules and under pressure, which have shown much higher iodine solubilities in glass (Riley et al., 2014; Jolivet et al., 2020). Iodine behavior within the melter can be driven by the feed composition, redox conditions, and operational variables (Bruffey et al., 2015). Within the melt, convection will move the iodine, and then iodine is limited by transport in the gas phase (Crichton et al., 1995). If a secondary phase containing iodine is formed, the buoyancy of that phase will dictate volatility. Salts will form a low-viscosity phase in the melt that will rise to the surface due to its low density and upon crystalizing the salt layer can segregate to its own phase (Jantzen et al., 2004). Small melt pools will have low overall mass transfer resistance for iodine to migrate to the melt surface (Choi, 2020).

The smaller halides, Cl and F, commonly have better retention in the glass compared with iodine but their presence in glass does not dictate iodine retention levels (Kruger et al., 2011). The initial hypothesis of lower solubility of iodine compared to other halides specified the atomic radii of the smaller halides can substitute for oxygen in the silica tetrahedra while iodine cannot (Crichton et al., 1995). However, this trend of decreasing atomic radii and improved retention has not always been observed (Shade, 1996). Recent work has suggested that Na and Li play a crucial role in stabilizing iodine within the glass as iodine retention increased with higher combined Na + Li content and the association of these cations with iodine in the glass network (McKeown et al., 2015; Jolivet et al., 2020; Vénague et al., 2022), although not strongly bonded (Muller et al., 2014). Contrary observations of increased iodine retention with lower Na content have been made (Kruger et al., 2011). The presence of iodine in glass can also affect the structure of the glass as polymerized glasses have been observed to depolymerize due to iodine and depolymerized glasses have begun to polymerize due to iodine (Jolivet et al., 2020).

Higher B content has been observed to increase iodine retention compared with Si-rich glasses due to viscosity limiting I diffusion and B-rich glasses typically having more alkali species available to stabilize Na–I (Cicconi et al., 2019a). The presence of Cs has also been shown to improve iodine retention by ∼60 mass% and the addition of K has improved iodine retention to a lesser extent (Kruger et al., 2011; Cicconi et al., 2019b).

Carbon-based reductants (sugars) additions to glass melts have demonstrated improved iodine retention in the melter. Combining sugar with another reductant facilitated >50% iodine retention (DF ∼2). However, a limit likely exists on sugar addition as increasing the stoichiometric carbon ratio was shown to generate an over-reduced glass (Kruger et al., 2011).

Several examples of iron improving iodine retention have been observed (Kruger et al., 2011). The best iron-based improvements to iodine retention were observed with the use of Fe(II)-oxalate where the reduced Fe(II) atom is active. The Fe(II) added serves as a reductant and hypothetically reduces the nitrate/nitrite present and improves iodine retention as a result. The use of zircon, garnet, and V_2_O_5_ have also been observed to improve iodine retention possibly through the same mechanism.

Iodide is the primary species identified in glasses containing iodine (Muller et al., 2014; McKeown et al., 2015; Gin et al., 2017), however iodate has also been observed (Riley et al., 2014; Cicconi et al., 2019a; Jolivet et al., 2020) and is suggested to have higher stability (Morizet et al., 2022). No clear trend on the resulting iodine species in the glass based on feed species (I^−^ and/or IO_3_^−^) has been observed (Muller et al., 2014; McKeown et al., 2015). Iodine speciation may also influence melter behavior as in melter testes differences in iodine retention were not observed when iodide or iodate was added to the melt, but CH_3_I was not retained in the glass (Kruger et al., 2018; Choi, 2020; Dixon et al., 2020).

If the iodine is retained in the glass, any release would be dictated by the dissolution of the glass network. Glass is well documented for its durability and a recent comparison showed a representative nuclear waste glass to have similar silicon dissolution rates compared to other iodine wasteform types (Reiser et al., 2022). With the maturity of vitrification technology and continuing enhancements on iodine retention, borosilicate glass will remain a viable option for the creation of iodine wasteforms.

#### 3.1.2 Silver phosphate glass

Silver phosphate glasses are expected to be vitrified in methods similar to borosilicate glass vitrification, although the glass would be vitrified at lower temperatures and the iodine source would be stabilized AgI. Silver phosphate glass has been used industrially in dosimetry (Hsu et al., 2010). The AgI–Ag_2_O–P_2_O_5_ system, first evaluated in 1999 (Fujihara et al., 1999) and 2008 (Sakuragi et al., 2008) has seen more recent interest since 2014 (Lemesle et al., 2014). However, pure silver phosphate glasses have a low glass transition temperature (*T*_g_) of <100°C, which is impractical for wasteform use. Therefore, additives can be used to increase *T*_g_ to >100°C, such as Al_2_O_3_ (Lemesle et al., 2014). This change in *T*_g_ occurs because the alumina acts as a crosslinking agent between the phosphate units. Essentially, the Al_2_O_3_ replaces P–O–Ag with a more chemically durable P–O–Al linkage (El Damrawi et al., 2020). In addition to Al_2_O_3_, Nb_2_O_5_ and Bi_2_O_5_ have also been investigated as a crosslinking agents (Chabauty et al., 2021, 2019). These additives are important to explore because using Al_2_O_3_ leads to formation of crystalline Al(PO_3_)_3_ phases, which limit potential iodine loading. Both Nb and Bi increase the stability of the glass but increase bond lengths which occur when incorporating iodine.

The matrix elements of silver phosphate glasses appear to have relatively high leach rates compared to iodine during Product Consistency Testing (PCT) and to other wasteforms (Yang et al., 2017a; 2017b; Reiser et al., 2022). Studied crosslinking reagents studied have had little effect on the chemical durability of phosphate-only glasses. However, a P_2_O_5_–MoO_3_ glass has also been investigated as molybdate may help form a passivating layer. With an Nb_2_O_5_ additive to P_2_O_5_ glass, a passivating layer was apparent (Chabauty et al., 2021, 2019). For phosphorous, the release rates decreased by two orders of magnitude and were dependent on Nb_2_O_5_ concentration.

**FIGURE 2:**
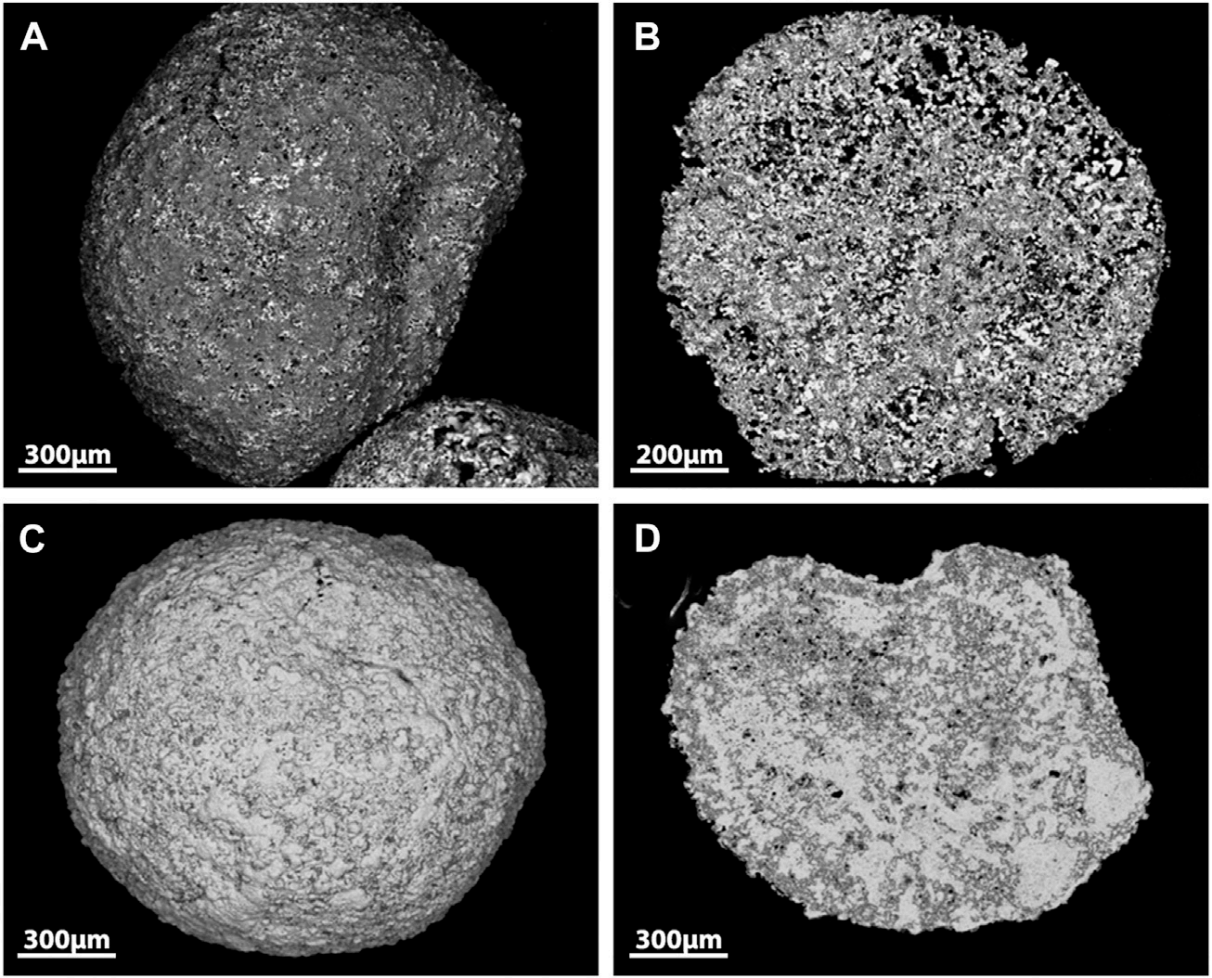
SEM images of Ag-phosphate glass sorbent surface **(A)**, sorbent cross section **(B)**, Ag-phosphate glass sorbent surface after I_2_ capture **(C)** and after I_2_ capture sorbent cross section **(D)** (Pénélope et al., 2022b).

An example of capture and wasteform integration has been reported for phosphate glasses (Figure 2) (Pénélope et al., 2022b). The sorbent comprised three crystalline phases: metallic silver, Ag_4_P_2_O_7_, and CaAg(PO_3_)_3_. The silver within the sorbent was shown to react with I_2_ and could then be converted into a AgI–Ag_2_O–P_2_O_5_ glassy system at 650°C. However, the authors noted that a separate AgI phase was also present in the wasteform indicative of an overloading of iodine. Durability data has yet to be presented. Compared to borosilicate glasses, the use of phosphate glasses is less widely applied and more challenging due to the low *T*_g_, but with further development, it could be realized as an industrially applied iodine wasteform.

#### 3.1.3 Lead-borate glass

**FIGURE 3:**
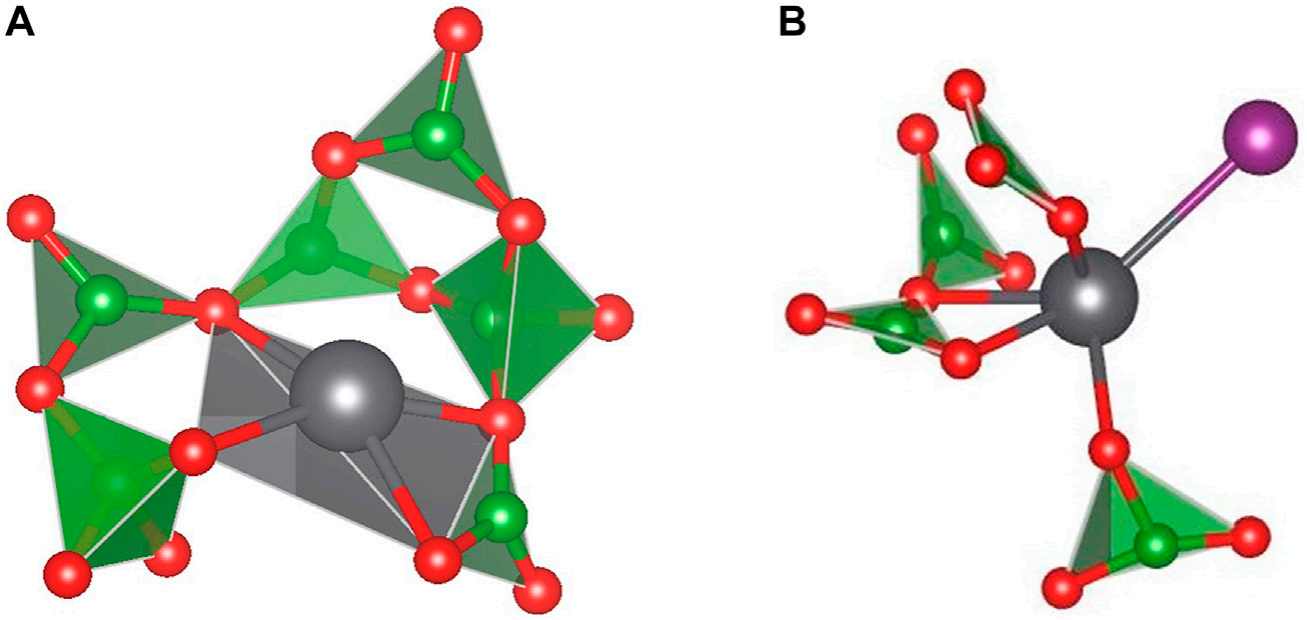
Typical structure of lead borate glass, showing Pb coordination **(A)** and Pb-I binding **(B)**. Grey: lead; Green: boron; Red: oxygen; Purple: Iodine (Mukunoki et al., 2016).

Lead-borate glasses would be prepared in methods similar to other glasses and have been proposed as a wasteform for iodine due to their relatively durable matrices and the low temperatures required for vitrification (Mukunoki et al., 2013, 2009, 2007; Tanabe et al., 2010). During the preparation of a lead borate wasteform for immobilizing iodine, H_2_ or another reductant would be used to remove the iodine from the sorbent followed by contact in a column with BiPbO_2_NO_3_ to generate a BiPbO_2_I compound (Mukunoki et al., 2016). This BiPbO_2_I is soluble within the glass matrix, and a recent study detailing structural information of these glasses has been reported using X-Ray absorption spectroscopy and modelling (Mukunoki et al., 2016). The glasses analyzed contained a modest 2.0 mass% iodine loading. The results indicated the iodine sits within the glass structure bound to the Pb and induces a change in coordination structure about the lead (Figure 3). Chemical durability studies have indicated that increasing PbO content leads to a decrease in durability, driven by incongruent dissolution of the matrix and the formation of a Pb-containing phase (Erdogan et al., 2014; Mukunoki et al., 2018). However, as lead-borate glasses are developed for iodine, the potential toxicity of lead must be kept in perspective for industrialization and disposal. This technology is continually being matured in pursuit of the disposal of reprocessing wastes, however it is less mature than borosilicate glasses.

#### 3.1.4 Tellurite glass

**FIGURE 4:**
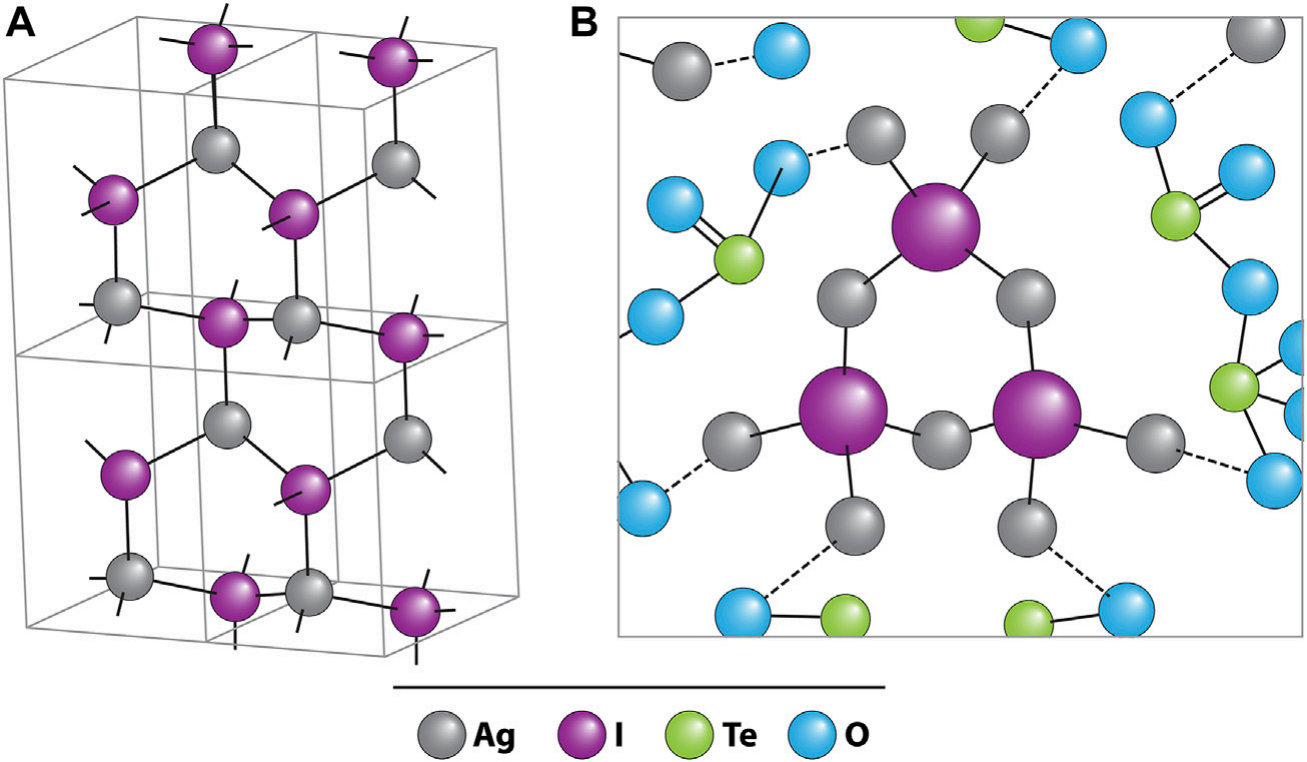
Schematic drawings of **(A)** β-AgI crystal in wurtzite structure; black balls: Ag^+^, open balls: I^−^. **(B)** Three [Ag_4_I] units sharing bridging Ag^+^ ions in silver tellurite glass; gray circles: O^2−^ (Lee et al., 2017).

Tellurite glasses (i.e., made with TeO_2_) have seen interest as iodine wasteforms because they can readily incorporate iodine into their glass structure. In recent years, a series of studies focusing on tellurite glasses have been reported, because of their potential for high loadings of iodine, good chemical durability, and low vitrification temperatures (reducing AgI volatilization potential). They are typically made by mixing solid precursors followed by vitrification with an emphasis on AgI due to its formation in common capture materials. For a 41TeO_2_–26Ag_2_O–11BiO_3_–22AgI glass made at 700°C, the AgI incorporated into the glass structure as a Ag_4_I unit chemically bound to the glass *via* non-bridging oxygens within TeO_3_ (Figure 4) (Lee et al., 2017). Additionally, the study observed that Te leaches around two orders of magnitude faster rate than I, showing that the Te phase may be sacrificial.

As in the case of other glass wasteforms, the use of crosslinking agents such as Bi_2_O_3_, Al_2_O_3_, and PbO has been investigated to try and reduce the breakdown of the tellurite glass network observed during leach studies (Kang et al., 2020). When the mass fraction of Bi_2_O_3_ and PbO are 10%, an order of magnitude reduction in Te release rates was observed.

In addition to common glass additives, transition metal oxides have been investigated (Kang et al., 2021). WO_3_, MO_3_, and V_2_O_5_ were added and led to similar order of magnitude reductions in the leach rate of Te. MoO_3_ or WO_3_ additions raise the glass transition and crystallization temperature of the resulting glass, whilst Bi_2_O_3_, and Al_2_O_3_ decrease it (Kang et al., 2022).

Under idealized conditions in pure water, iodine leaching from these materials can be as low as 10^−7^ g∙m^−2^∙d^−1^, which puts the material amongst the best performing wasteforms to date (Reiser et al., 2022). Tests using a Bi_2_O_3_-doped tellurite glass were carried out in acidic leach (0.1 M acetic acid) conditions where iodine leached 10 times faster than in deionized water (Lee et al., 2021). Similar to other proposed vitrified wasteforms, increasing maturity is ongoing but industrial application is not ready as of yet.

### 3.2 Glass ceramics

Glass ceramics differ from glasses in that the wasteform contains a mixture of phases, typically an amorphous phase and at least one ceramic phase but potentially multiple. The differentiation made is that the iodine-containing phase must be encapsulated by the glass phase rather than chemically incorporated into the glass structure. If the glass phase has higher durability than the iodine-containing phase, the glass phase can protect the iodine phase. Glass ceramics are suitable for stabilized iodine but can also handle liquid-fed iodine. Significant work, discussed below, has focused on developing materials that can be made at a low temperature to avoid iodine volatilization during vitrification whilst ensuring that the resulting material is durable. This durability is achieved by minimizing the resulting porosity of the wasteform and by incorporating additives that provide crosslinking to stabilize both phases. By changing both the synthesis conditions and additives, formulation properties can be optimized. However, a main challenge toward industrialization is the implementation of this low-temperature ceramic process while ensuring adequate full-system processing.

#### 3.2.1 Boron glass ceramic

Boron glass ceramics have been explored as a potential wasteform for iodine (added as stabilized iodine) due to the low melting point of boric acid. Boron oxide, silica, and AgI powders were heated to between 450–550°C to create B_2_O_3_–AgI–silica gel wasteforms with loadings of iodine up to 8.7 mass% (Wei et al., 2019). Synthesis temperature and silica loading were varied to identify a material that had the highest fraction of amorphous material and the lowest porosity. The maximum amorphous content (98%) was achieved when the loading of AgI-silica was at the lowest ratio investigated (20 mass% AgI–80 mass% silica) whilst the temperature was at its highest (550°C). These conditions were also used when the porosity was found to be at its lowest (10.6%). No durability data was presented.

To control the properties of a boron glass ceramic, ZnO and Bi_2_O_3_ have been added to aid in formation of separate BO_4_ and ZnO_4_ glass units along with the stabilized Ag. Bi_2_O_3_ can form BiO_*n*_ (*n* = 3 or 6), which can combine with B_2_O_3_ to favor formation of BO_4_ units which increase glass stability (Litwinek-Rozbicka et al., 2021; Feng et al., 2022; Wen et al., 2022). ZnO reduces the glass transition temperature and increases the density of the glass (Kim et al., 2009). Over a series of studies, which typically involved iodine in the form of AgI, the level of amorphous material and the final density were investigated (He et al., 2022; Wang et al., 2021; Wei et al., 2020; 2021a; 2021b; Meng Yan et al., 2021; J. Yang et al., 2021a). The authors used density and amorphous content as a proxy for durability and found that in general:

• Bi_2_O_3_: For each study where this variable was changed, 1 mol% (M. Yan et al., 2021b), 10 mol% (Wang et al., 2021), and 20 mol% (Wei et al., 2020) were found to contain the highest proportion of amorphous material out of their respective studies. The implication is that, in general, a lower fraction of Bi_2_O_3_ is preferable in the formulation.
• ZnO: Without ZnO, formation of a wasteform was possible, but there was evidence of undesirable crystalline AgI phases as well as soluble iodide (M. Yan et al., 2021b).
• Temperature: Across the studies, higher temperatures tended (up to 600°C) to lead to greater amorphous fractions and a more homogenized AgI distribution (Wei et al., 2016).
• Precursor synthesis method: By changing the way in which the precursor pellet was created, prior to sintering, the final density of the sintered material could be increased.
• AgI:silica loading: Across the studies, 10–30 mass% was investigated. In general, lower AgI: silica loadings were preferable to yield an amorphous product with AgI homogeneously incorporated.

The silica from the AgI loaded capture material plays a role in forming the glassy phase. In some of the studies, the impact of SiO_2_ on the properties of the wasteform was investigated (Wei et al., 2021c; Meng Yan et al., 2021; Wu et al., 2021, 2022; Yuan et al., 2021). This included adding in additional SiO_2_ to further alter the properties. A key takeaway from this work was that starting from an amorphous form of SiO_2_, such as a silica gel rather than crystalline SiO_2_, led to more amorphous material after low-temperature sintering (Wei et al., 2021c). The formation of separate AgI phases within the wasteform as the iodine loading increases is challenging to avoid, as can be observed across a number of studies (Wu et al., 2021; M. Yan et al., 2021a; Yuan et al., 2021). These results show promise for the use of glass ceramics for iodine wasteforms but are at an early stage of development with respect to industrialization and understanding durability.

### 3.3 Glass composite materials

Glass composite materials (GCM) are heterogeneous and contain both crystalline and/or glass phases. They differ from glass ceramics in that the crystalline phases play a role in the stability of the wasteform with the glass material acting as a binder, which increases durability. To date, development has been limited to the lab scale. Examples include the used of Bi–Si–Zn oxide glass to bind with AgI-loaded mordenite (AgZ + I) with additional Ag to capture any I_2_ released during processing (Garino et al., 2011). The resulting GCM was more durable than just AgI-loaded mordenite (Mowry et al., 2015). GCMs extend beyond zeolites, with examples of metal organic frameworks (MOF) being combined with a Bi–Zn oxide glass (Sava et al., 2012) and a mesoporous silica (SBA-15) combined with Bi_2_O_3_ glass (Yang et al., 2015).

More recently, a GCM was produced with the aim of creating iodosodalite phases (Liu et al., 2021). Here, 5–25 mass% of a Bi_2_O_3_ glass powder was mixed with zeolite-4A and NaI and sintered at 500–1100°C. Sintering temperatures of 600°C were found to be ideal to avoid NaI decomposition along with 15% glass powder. The presence of iodosodalite was confirmed by X-ray Photoelectron Spectroscopy (XPS) along with homogenous iodine distribution, which may help improve chemical durability. The chemical durability of the GCMs were studied by 7-day PCT in near-neutral conditions and were similar to other glass-bonded sodalites.

### 3.4 Low-temperature hydrating or polymerizing wasteforms

If chemical durability can be demonstrated, the advantages of cementitious matrices make them an interesting wasteform candidate. Advantages include low-temperature processing and using already established waste processing techniques. In general, these wasteforms are made by mixing dry reagents with an aqueous solution to initiate hydration reactions, generating a hardened material. The iodine could be present in several forms, as a dissolved species, as stabilized AgI, or present in the initial liquid. Iodine has complex behavior within cementitious matrices as its species can both sorb and incorporate to the matrix phases (Toyohara et al., 2000; Kaplan et al., 2019; Nedyalkova et al., 2020; Gillispie et al., 2021; Fujii Yamagata et al., 2022). The sorption process has been found to be proportional with an increasing Ca:Si ratio in the wasteform. Iodate, with its outer oxygen atoms, has stronger affinity for cement matrix phases than iodide (Evans, 2008). The retention of iodine within cement matrices has been shown to improve when the iodine species precipitated into a low solubility salt, i.e., AgI (Burger et al., 1981). However, reliance on the low solubility of AgI for maximizing chemical durability may not be appropriate in reducing environments. If the redox chemistry remains favorable, then it seems practical that inclusion of an iodine-loaded sorbent (AgZ) would have a low release of iodine from the cement wasteform.

Some of the earliest work on investigating iodine-loaded sorbents in cementitious matrices was performed in the early 1980s (Burger et al., 1981). In this work, a comparison between AgZ + I in a cementitious wasteform and Ba(IO_3_) in cement (BaIO_3_ cement being the baseline at the time for the byproduct of the Mercurex process) showed that iodine was released an order of magnitude slower from the AgZ-cementitious wasteform (Trevorrow et al., 1983). However, when AgI was added directly the iodine release was even lower. An iodine-loaded Pb-zeolite was also investigated but only showed limited improvement in iodine release due to hydrolysis of the PbI_2_.

At the U.S. Hanford site, reduced AgZ is planned to be used to control releases of radioiodine during the vitrification of high-level waste (Scheele and Wend, 2015). Two research efforts have been presented to study the stabilization of the spent iodine-loaded AgZ (Scheele and Wend, 2015; Fujii Yamagata et al., 2022). In the Scheele work, wasteforms with 25 mass% AgZ were prepared using Portland Type III cement (65 mass%) modified to include calcium iodide (10 mass%) to control silver release due to its toxicity. Analysis showed that in US Environmental Protection Agency Method 1311, the wasteform was regulation-compliant for silver release, showing the effectiveness of CaI_2_ as a modifier to improve Ag retention.

**FIGURE 5:**
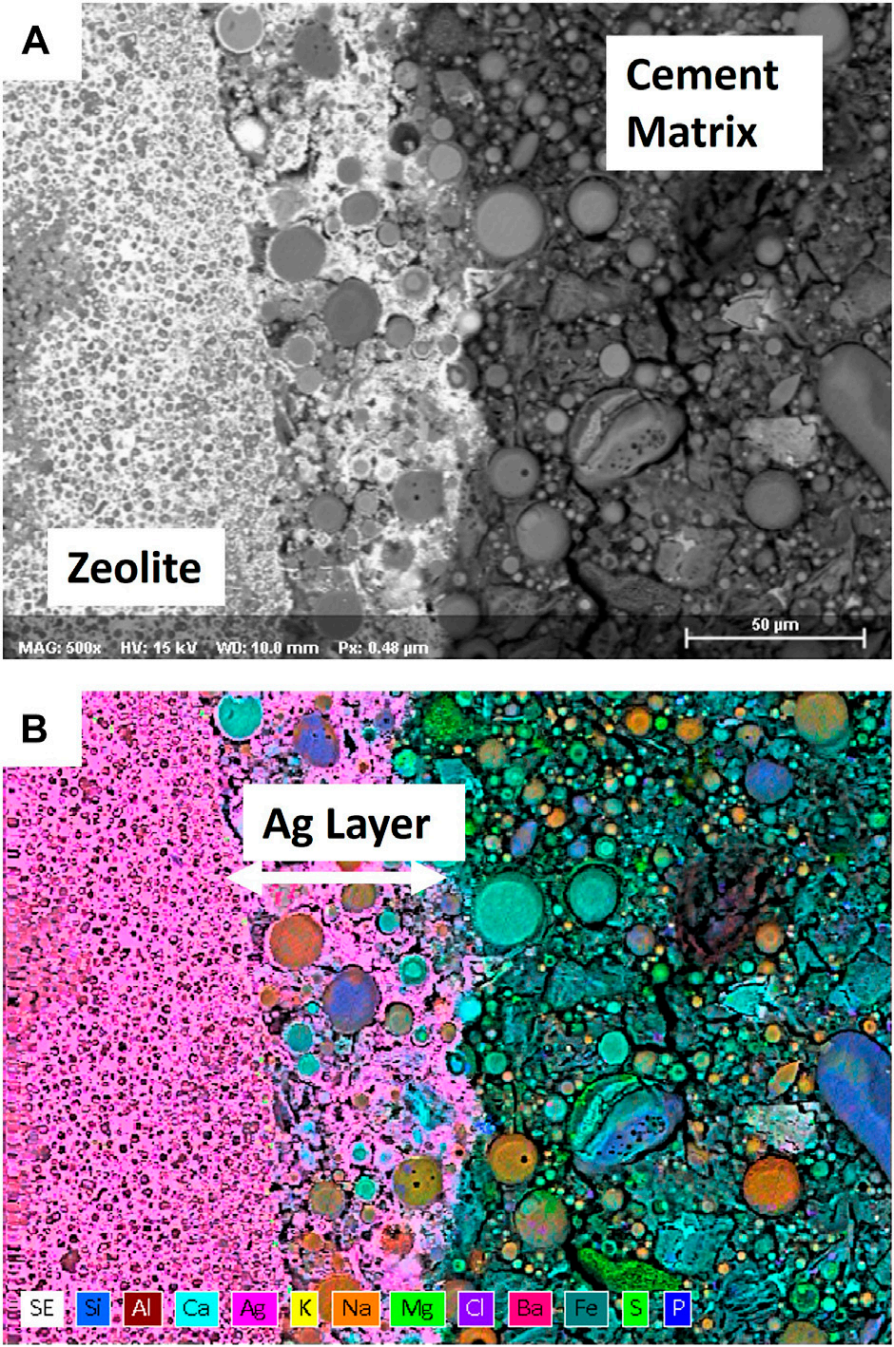
Scanning Electron Microscopy (SEM) micrograph **(A)** and corresponding Energy Dispersive X-ray Spectroscopy (EDS) map color map **(B)** of an Ag-enriched region between an Ag-zeolite particle that had been encapsulated in a cementitious matrix. Samples were prepared in a method similar to the method described in (Yamagata et al., 2022).

A recent effort demonstrated that AgZ + I can be successfully immobilized in three different cementitious formulations under consideration at the Hanford site with little impact to wasteform mechanical properties up to 30 vol% (Fujii Yamagata et al., 2022). Semi-dynamic leach testing of the AgZ containing wasteforms showed that in an oxidized formulation (no blast furnace slag included), no iodine was detected in the resulting leachates. This measurement was similar to another test where 5 mass% AgZ was added to a cementitious wasteform for iodine in liquid waste and on stabilizing silver alumina, silver silica gel, and AgZ in Portland cement (Mizuno et al., 1986; Lockrem, 2005; Crawford et al., 2017). This enhanced iodine retention was attributed to an Ag-layer that was observed to form at the interface between the AgZ particle and matrix, see Figure 5. The Ag-layer could act as a barrier to limit iodine migration from the AgZ to the cement matrix (Fujii Yamagata et al., 2022). In samples containing blast furnace slag to create reducing conditions, some iodine release was measured from the wasteforms, and was likely due to reductive dissolution of the AgI or competition from sulfide. Testing of iodine-loaded metal-functionalized silicates in geopolymer matrices showed the presence of Ag_2_S and a breakdown of the silica backbone under the high-pH conditions used to create the geopolymer (Kearney et al., 2022). This behavior has also been observed when studying AgZ immobilized in both slag-containing and slag-free wasteforms (Kaplan et al., 2019). A 60-day batch experiment demonstrated that AgZ in slag-free grout was extremely effective at immobilizing I and Ag while a grout containing slag was less effective. Both grout samples showed the iodide originally present on the AgZ was leached primarily as iodide and organo-iodide, with more organo-iodide in the slag-free system. These results highlight that subsurface disposal of grouted AgZ should be done under oxidizing conditions and that species transformations are possible.

The use of cementation to stabilize iodine-loaded sorbents has also extended to activated carbons. Ag-impregnated activated carbon (Ag-GAC) has been studied when stabilized in cementitious materials (Li et al., 2019). The Ag-GAC embedded in cementitious material was very effective in sequestering iodide and organic-iodide in cement porewaters suggesting it can hold iodine upon disposal. Any I that was released from AgI was in the form of iodide.

This work shows speciation within cement-based wasteforms should be monitored during the conversion from sorbent to wasteform as it is crucial to understanding long-term disposal. In all, cementation and other low-temperature processes are very mature and applied widely at the industrial scale. Minimal handling is required to add stabilized or soluble iodine to a cementitious matrix. However, disposal site acceptance criteria may limit the use of these cementitious wasteforms for iodine disposal.

### 3.5 Inorganic minerals and ceramics

#### 3.5.1 Sodalite

Iodosodalite [e.g., Na_8_(AlSiO_4_)_6_I_2_] is a highly durable wasteform attracting a significant amount of continued interest. In particular, recent developments include further details on the use of glass binders to enable densification at 750°C (Chong et al., 2020, 2018), the use of cold sintering to potentially eliminate the need to use glass binders (Hassan et al., 2020), the use of sol-gel methods (Kroll et al., 2020), the immobilization of AgI (Kuo et al., 2018; Maddrell, 2019), the interzeolite conversion to sodalite (Liu et al., 2021, 2020; An et al., 2020), and the direct conversion of waste streams into sodalite (Neeway et al., 2016; Bollinger et al., 2022).

Glass binders have been proposed to be used in the consolidation of sodalite to reduce porosity, promote sodalite formation, and reduce formation of unwanted crystalline nepheline or quartz phases. Common glass binders include NBS-4 and SA-800 and further details of the impact of varying temperature and glass loading were reported (Chong et al., 2018). In a recent report, direct sodalite synthesis was evaluated by reacting zeolite 4A (i.e., Na_12_Al_12_Si_12_O_48_) with NaI or AgI (Riley et al., 2019). For this process, NaI or AgI were occluded into zeolite 4A at 500°C followed by the addition of a glass binder (NBS-4) at 26 mass% and heat treatment at 925°C in a glassy carbon crucible. The target sodalite phases for the NaI and AgI samples were Na_8_ and Ag_2_Na_6_ sodalites. However, other work shows that creating a mixed cation sodalite (e.g., Na + Ag) is not a straightforward process and might involve a unique set of processing conditions (Maddrell et al., 2015).

Aqueous synthesis of iodosodalite can be performed by starting from NaI solution and mixing this with other additives like NaOH, metakaolin (Al_2_Si_2_O_7_), kaolinite [Al_2_Si_2_O_5_(OH)_4_], or NaAlO_2_ + colloidal silica (Chong et al., 2018; Nam et al., 2018). These reactions can proceed at low temperatures at atmospheric pressures in open containers or at elevated pressures in autoclaves. As-synthesized materials have been mixed with glass binders and fired at temperatures of 650, 750, or 850°C to yield wasteforms with lower porosities (Chong et al., 2018). In studies like this, finding the optimum sintering temperature is important because insufficient temperatures will often result in more open and closed pores (higher void space volume), whereas firing at too high of temperatures can lead to sodalite decomposition (e.g., nepheline formation) and higher amorphous fractions (Riley et al., 2021).

The optimal sintering temperature (750°C) for these materials enables the consolidation process whilst avoiding substantial iodine volatilization. Here, NBS-4 and SA-800 led to different crystalline fractions, with NBS-4 leading to more crystallization. However, the proportion of sodalite was lower for SA-800. PCTs from NaI-based iodosodalite showed low cumulative iodine releases of 2 × 10^−5^ g∙m^−2^∙d^−1^ after 28 days (Chong et al., 2018). Chemical durability could be further improved through reductioChongn of residual pores in these products altering process conditions.

In follow up work, HIP was used as the consolidation method for iodosodalites and also focused on comparing the impact of the sodalite synthesis on the wasteform durability (Chong et al., 2020). Sodalite synthesized by an aqueous method had a lower porosity (1.2 vol%) than a hydrothermal equivalent (2.2 vol%). Correspondingly, a leach rate 4 times higher for a sample synthesized *via* the hydrothermal method was recorded (Reiser et al., 2022). In addition, the aqueous method led to reduced formation of nepheline and volatilization of iodine during HIP consolidation. Furthermore, synthesizing sodalite prior to consolidation by a process such as HIP led to a more durable wasteform with fewer unwanted phases and lower iodine volatilization.

**FIGURE 6:**
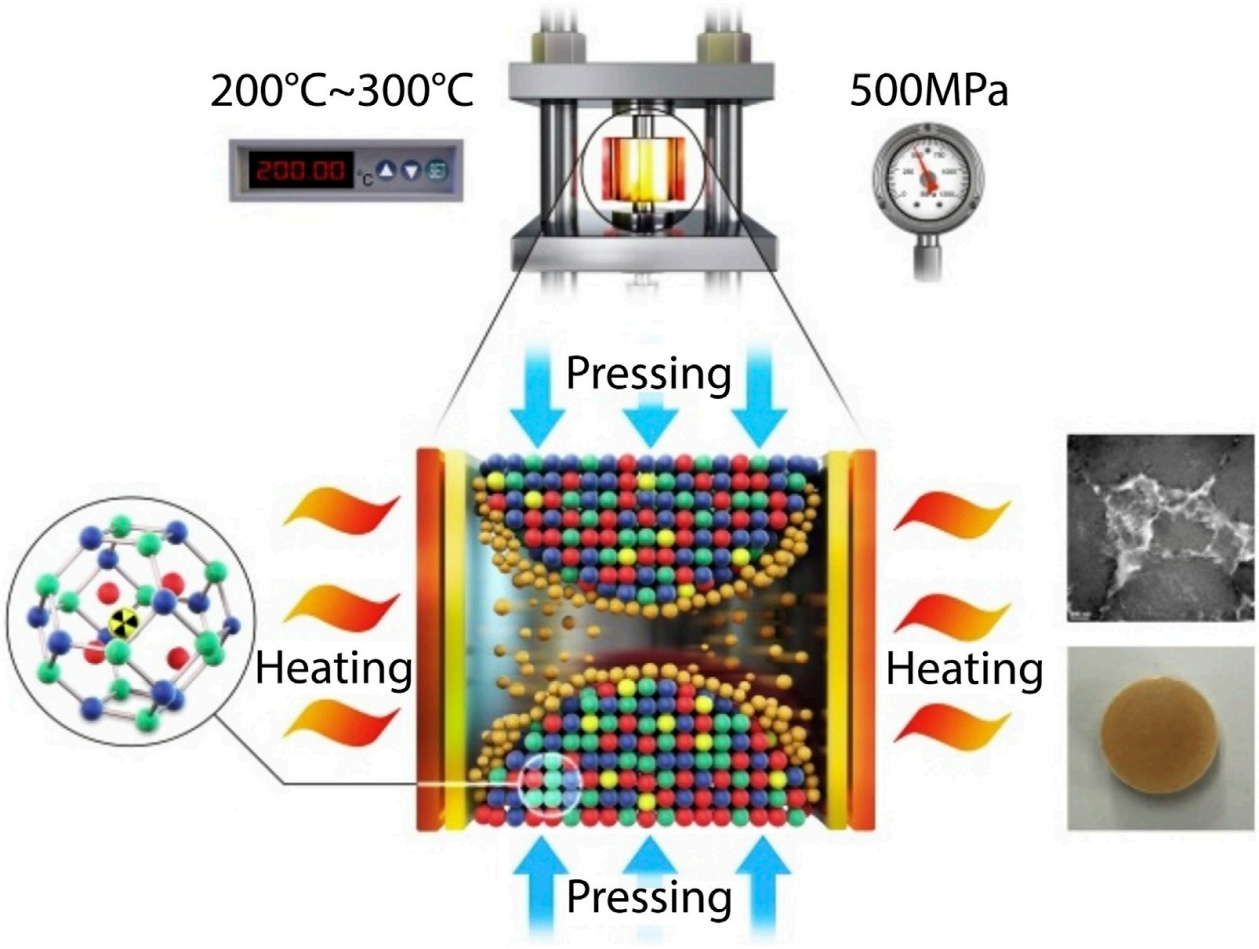
Schematic of cold sintering process to consolidate iodosodalite (Hassan et al., 2020).

The challenge of avoiding iodine volatilization and formation of crystalline phases can potentially be avoided by the use of cold sintering (Figure 6), which offers a method of keeping temperatures low (200–300°C, 500 MPa) during consolidation of sodalite (Hassan et al., 2020). Keeping the temperature below 300°C was found to avoid iodine loss. Up to 91% densification could be obtained at 500 MPa and 300°C. The densification is postulated to be achieved in two steps: 1) compaction through sliding and rearrangement of crystalline and amorphous phases under the applied pressure and 2) further densification from the dehydration of the amorphous phase. Hydrothermally synthesized sodalites, which were used in the Hassan et al. (2020) study, are expected to have an amorphous phase that is >40 mass% of the material. Leach data presented was comparable to consolidation of sodalite in the presence of glass binders by HIP.

Another alternative for iodosodalite synthesis reported is a sol-gel synthetic technique used to reduce the temperature needed for sodalite formation (Kroll et al., 2020). It has been suggested that the resulting gel is suggested to may then be converted into a dense sodalite solid using higher temperature heat treatments. The gels were created at room temperature from varying mixtures of Si(OC_2_H_5_)_4_, Al(OC_4_H_9_)_3_, B(OC_2_H_5_)_3_, NaOCH_3_, NaOC_2_H_5_, and NaI. In some cases, 10 mass% NBS was added to allow iodosodalite formation at lower temperatures. After drying the gels at 90°C, they were then ground into a powder, pressed into a pellet and sintered at temperatures between 450–900°C.

Whilst the majority of reported synthetic methods for sodalite require iodine in the form of NaI, other studies used AgI as the starting material. Reports include the conversion of a silver exchanged faujasite (IONEX Ag-400) into a silver sodalite (Maddrell et al., 2015) by HIP and attempts to synthesize AgI-sodalite and consolidate by HIP (Vance et al., 2016). The interzeolite transformation of AgI loaded zeolites did not lead to 100% conversion to sodalite, with some AgI phase remaining in iodine-rich areas of the zeolite. During the HIP process, in-can interactions between the wasteform and Cu/Ni (Maddrell, 2019) and Fe (Maddrell et al., 2015) HIP canisters have been observed up to 200 μm in the matrix leading to enhanced release of iodine. The AgI-sodalite was also shown to be less durable under reducing conditions, consistent with results from other AgI-based wasteforms, again showing the promise of NaI-based waste forms in reducing conditions. Mixed Na/Ag clusters appear to not have formed, instead discrete Na_4_I and Ag_4_I moieties are present. This experimental observation is supported by calculations that reveal an energy penalty for having both Na and Ag in the cation structure (Na_*x*_Ag_4-*x*_I) (Kuo et al., 2018).

Interzeolite transformation describes the process where a zeolite structure can be converted into a different one, in this case to a sodalite and another example of where a material designed for iodine capture is better to be converted with a final wasteform. Zeolites may convert into sodalite using a hydrothermal method (An et al., 2020). Hydrothermal methods work better for NaI-loaded zeolites rather than AgI-loaded zeolite (Maddrell, 2019). In addition, when the Si:Al molar ratio of the zeolite is 1, the interzeolite conversion leads exclusively to sodalite. When Si:Al is increased to 1.2, sodalite is formed in addition to zeolite ANA [ANA = (AlSi_2_O_6_)^−^] (Wang et al., 2010). When Si:Al is increased further to 2.6, no sodalite can be formed by this method. Of all the materials tested, the Na-X zeolite starting material had the best compromise between wasteform properties and iodine capture performance. Around 120 mg/g (not including physiosorbed iodine) of CH_3_I was found to react with the zeolite to form NaI of which ∼98% was immobilized in the sodalite cage during interzeolite conversion.

The use of an additive can potentially aid the interzeolite transformation. Mixing NaI with Zeolite-4A (Si: Al = 1) in the presence of B_2_O_3_ can yield sodalite after sintering at 750°C (Liu et al., 2020). The B_2_O_3_ promotes the formation of iodosodalite at a lower sintering temperature. However, temperatures of 1050°C led to a higher fraction of iodosodalite but likely at the expense of iodine volatilization. For the sodalite formed at 1050°C in the absence of B_2_O_3_, a low iodine leach rate under PCT conditions (10^−5^ g∙m^−2^∙d^−1^) was reported. Similar durability has also been observed for gas pressure sintered iodosodalites.

Bi_2_O_3_ has also been investigated as a glass powder additive to promote sodalite formation (Liu et al., 2021). The advantage of Bi_2_O_3_ over B_2_O_3_ is that it reduces the sintering temperature required to convert to sodalite to 600°C, below the NaI decomposition temperature of 670°C. The PCT leach rates of samples using Bi_2_O_3_ additives were 10^−3^ g∙m^−2^∙d^−1^, consistent with other glass-bonded sodalites. The synthesis reported requires a ball milling step to ensure that the particles are well mixed, but it does avoid the need for hydrothermal synthesis.

Another approach is to take a waste stream that includes more than iodine and to convert everything into an individual wasteform. In an example of a wasteform that immobilizes more than just iodine, sodalites have been shown to form during fluidized bed steam reforming (FBSR) such as the process used at Idaho National Engineering Laboratory (Neeway et al., 2016). Once processed, the resulting wasteform contained mostly nepheline, nosean, and sodalite. Leach experiments using the ASTM single-pass flow-through (SPFT) test were on the order of 10^−4^ g m^−2^∙d^−1^ at pH 9. Another example involves the conversion of spent caustic scrubber liquors used to capture C-14, NO_*x*_, and I-129 in off-gas streams in reprocessing into a sodalite-based wasteform (Bollinger et al., 2022).

Sodalites remain a promising candidate for iodine wasteforms due to the high durability offered by the NaI-sodalite. However, significant challenges remain. Firstly, AgI-sodalites appear less durable in potentially reducing disposal environments, which may hinder attempts to directly convert capture materials into iodosodalite wasteforms. The alternative NaI-sodalite appears more durable but is less compatible with current capture material concepts, therefore requiring a more involved wasteform production process.

#### 3.5.2 Direct conversion of silver zeolite

Silver zeolites, including AgZ, faujasite (AgX or AgY), and Linde Type-A (AgA), have been developed for use in adsorbing iodine from nuclear reprocessing off-gas streams (with AgZ more widely studied) (Jubin and Strachan, 2015). Zeolite minerals are hydrated aluminosilicate minerals with a specific tetrahedral layered AlO_4_/SiO_4_ structure. The direct conversion of an iodine-loaded Ag-zeolite is desirable as it can make a high density, thermally stable, non-hazardous wasteform without the need for pre-treatment (Jubin et al., 2017). Recent work has targeted a better understanding of the processing and final wasteform product (Jubin et al., 2017), durability testing (Asmussen et al., 2019) and the effect of the HIP canister material construction on the wasteform (Maddrell, 2019).

The use of HIP for consolidating iodine-loaded silver zeolite or silver silica gel with a metal additive was originally patented in 1998 (Fujiwara et al., 1999). The development of the HIP process was followed by research showing that the heating of metal-doped forms of zeolites with AgI can lead to iodine transfer to the zeolite pores, and these occlusions can could generate a durable wasteform (Hyatt et al., 2003). HIP could be used to drive this process, but a temperature limit of 750°C was proposed based on the resulting product stability. AgI could also be protected in a TiO_2_ matrix produced *via* HIP (Maddrell and Abraitis, 2003). Further development demonstrated that the HIPing of silver zeolite with AgI salts generated consolidated waste forms of Ag-I sodalites. Some free AgI salts were observed in the preparation. Here, AgY was not observed to form sodalite, while AgA and AgX formed monolithic sodalites. Samples that originated with a AgA had higher initial iodine release, and the HIP versions of the zeolites were more durable than similar wasteforms containing a glass binder (Reiser et al., 2022).

Production of a “synthetic rock” wasteform through HIP in Japan has targeted an I-loaded alumina adsorbent having been pretreated to thermally convert AgNO_3_ and AgIO_3_ to Ag and AgI, respectively (Tanabe et al., 2010; Miyakawa et al., 2012). HIPing at a lower temperature of 200°C was used to generate wasteforms from AgX sorbents used for iodine capture during the oxidation of used fuel. Neutron radiography confirmed the sequestration of the iodine in the waste form (Westphal et al., 2010).

The most extensive work on HIP of AgZ has been carried out at Oak Ridge National Laboratory (ORNL) beginning in 2013. The first works toward conversion of iodine-loaded AgZ used hot uniaxial pressing (HUP), however the low pressure used (28 MPa) led to production of a fragile wasteform that only had a 70% density increase over the raw material. HIPing was then studied, in three testing phases, to assess any improvements to the wasteform. A study on the variation of temperature and pressure showed an improvement in densification of over 300%. An expanded matrix of samples was prepared to examine the effects of multiple source materials, compositional variations, and an expanded temperature range. Visual differences were observed, but the samples remained primarily amorphous with densifications of ∼300%. A follow-up study focused upon multiple mordenite forms, including sodium mordenite (NaZ), pure AgZ, and engineered AgZ mixed with a clay binder material. Through this matrix, the variables investigated included the pressure and temperature of pressing, the ratio of mineral to iodine, and the form of iodine (NaI vs. AgI) (Bruffey and Jubin, 2015). PCT results showed that iodine and sodium leached rates decreased substantially as pressing temperature was increased from 525°C to 900°C. Further increase of the pressing temperature to 1100°C did not result in measurable improvement in leach rates.

Further work was performed on the HIP AgZ to better understand the processing and final wasteform products (Bruffey et al., 2016; Jubin et al., 2017). The results showed that sodalite was confirmed in samples made using AgA but not AgX at both 175 and 300 MPa, while the addition of alumina did not help convert to sodalite in both samples. No difference in product form was observed with increased iodine content. Each type of zeolite produced a distinctive surface: chemisorbed samples were observed to have hairline fractures throughout the material, and occluded samples were observed to have small voids in the cross-sectioned surface. One of the most significant observations was that no iodosodalite formation was observed for any of the AgZ + I samples, either chemisorbed or occluded. This provides further confirmation of the importance of the Si:Al ratio for zeolite mineral behavior during HIPing. The samples prepared in this effort have been evaluated for durability using SPFT testing and semi-dynamic leach testing (Asmussen et al., 2019; Reiser et al., 2022). At low HIP temperatures of 525°C, the matrix of the HIP AgZ samples was more susceptible to corrosion attack, while AgZ samples HIPed at 900°C led to high initial iodine release and lower matrix dissolution but showed evidence of large host-phase (AgI) exposure with time.

**FIGURE 7:**
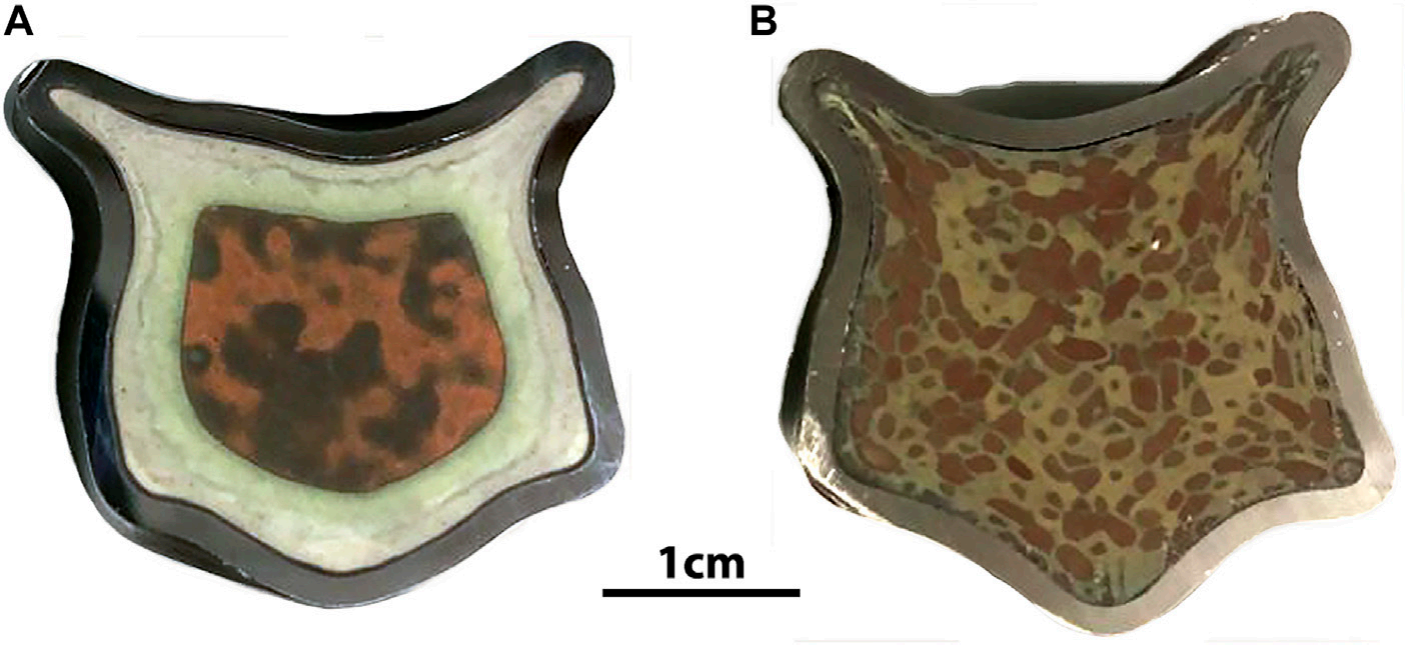
Comparison of the microstructure of large format HIP AgZ samples prepared identically except for the having iodine loadings of **(A)** ∼135 mg(I)∙g^−1^ AgZ and **(B)** ∼60 mg(I)∙g^−1^ AgZ. Preparation details in Reiser et al., 2022.

Scaled test samples of HIP AgZ have also been prepared. The HIP AgZ samples prepared at ORNL in the initial phases had an initial volume of ∼6.5 cm^3^ and larger samples of ∼57 cm^3^ were prepared. Two different sample pairs were prepared by HIPing at 900°C and 175 MPa using the same material but differing iodine loadings. The resulting wasteforms had strikingly different surface morphologies (see Figure 7). The higher iodine loading sample had a green ring comprised of large, isolated areas of AgI while the brown areas in both samples had smaller interconnected amounts of AgI. The microstructures did not match those from smaller samples produced at similar conditions. The durabilities of these samples are reported elsewhere where the large-form samples had higher iodine releases than comparative samples produced at smaller scales, although the matrix dissolution rates based on Si were comparable regardless of sample size. These findings highlighting the need to better understand the effect of scaling on iodine wasteforms.

A study of AgZ HIPed in steel canisters has shown that the corrosion resistance of the steel can be lowered due to galvanic contact with AgI (Ebert et al., 2019). When iodide is released from the wasteform, localized corrosion of the steel is postulated to be induced. This interaction requires further investigation and with different HIP canister metals. HIPing of AgZ, or other iodine capture materials, holds much promise, although has yet to be demonstrated at full scale.

As industrial HIP deployment increases in maturity, the attraction of a simple wasteform conversion process makes the high pressure and temperature conversion of AgI-loaded zeolites an attractive option for a consolidated wasteform.

#### 3.5.3 Apatite: Lead vanadates (PbV)

Of the apatite materials for iodine, lead vanadates [Pb_10_(VO_4_)_6-*x*_(PO_4_)_*x*_I_2_] have received the greatest interest over the years (Riley et al., 2016). The crystal structure of lead apatite is flexible, enabling a wide range of elements to be incorporated into the crystalline phase; however, the main drawback is the use of a toxic metal (Pb). The synthesis of lead vanadates requires iodine to be in the form of NaI, but work has been done to synthesize vanadates directly from AgI or PdI_2_ (Johnstone et al., 2020, 2017). Unfortunately, multiple phase materials were generated. When the PdI_2_ precursor was used, the phases included reduced Pd metal, PdI_2_ and Pb_9.85_(VO_4_)6I_2_. Starting from AgI, heterogeneous phase distributions of M_3_(VO_4_)_2_ (M = Ba, Pb) and AgI were formed.

SPS has been studied as a technique to consolidate the wasteform along with the durability of the resulting material that can contain up to 8 mass% iodine (Z. Zhang et al., 2018b; Zhang et al., 2019a; 2019b).

**FIGURE 8:**
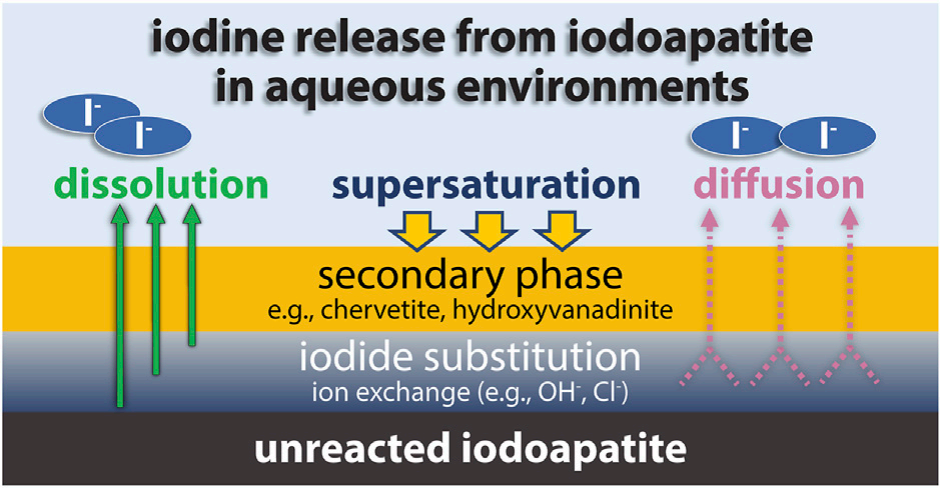
Schematic diagram illustrates major processes that control the iodine release from iodoapatite in aqueous environments, adapted from (Zhang et al., 2019b).

A more detailed understanding of the leach mechanism of iodine-containing lead vanadates has been uncovered in recent years (Figure 8). During leaching in the initial stages, hydroxide substitutes for iodide in the matrix by diffusion. Over time, this rate slows as the amount of iodide on the surface decreases (Z. Zhang et al., 2018b). When the pH is near neutral, this iodine diffusion process slows (Zhang et al., 2019a). At low pH, the release is controlled by the rate of matrix dissolution. In non-neutral pH, secondary phases can form that hinder the dissolution rate, but this effect on dissolution rate is not as significant as the effect of a maintaining a neutral-pH system. Higher ionic strength leads to accelerated dissolution as the activity coefficient of the reacting aqueous species is reduced (Zhang et al., 2019b).

A lead-arsenate apatite Pb_5_(AsO_4_)_3_I has been reported, although a durability study has yet to be carried out (Sordyl et al., 2020). The described synthetic approach is similar to the wet precipitation method for calcium phosphates. It involved dropwise addition at room temperature of Pb(NO_3_)_2_, Na_2_AsHO_4_·7H_2_O, and KI at a constant pH of 4.5. Lead vanadates have a pathway to industrialization but would likely require a conversion process of the iodine and a need to evaluate the impact of Pb toxicity.

#### 3.5.4 Apatite: Calcium phosphates

Iodate-substituted hydroxyapatites [Ca_10_(PO_4_)_6_(IO_3_)_*x*_ (OH)_2-*x*_] have been of interest for a few reasons. The crystalline lattice contains a high iodine content (8.4 mass%) (Guy et al., 2002), the material shows promising durability, and the calcium phosphate apatite avoids the need to use toxic materials (e.g., Pb). These materials require that iodine is in the form of an iodate, typically reported using KIO_3_/NaIO_3_/NH_4_IO_3_ as the precursor to apatite formation, which takes place by a wet precipitation reaction with Ca(NO_3_)_2_ and NH_4_H_2_PO_4_ (Campayo et al., 2011).

Recent work on these materials has focused on increasing consolidated material densitieswhich reduces the surface area that is exposed to leaching. Studies involving low-temperature SPS (Coulon et al., 2017, 2016) and cold sintering have been reported (Hassan and Ryu, 2019). The requirement for lower temperatures comes from both the stability of the apatite and volatility of the iodine. The durability of these materials has also been investigated in greater detail than has previously been reported (Coulon et al., 2017; Hassan and Ryu, 2019).

Densification using SPS could achieve up to 80% of the theoretical maximum densities of densified apatites at 150–250°C (Coulon et al., 2016). More recent work using cold sintering at 200°C and 500 MPa for 10 min improved this to 96.8% (Hassan and Ryu, 2019). No volatilization of iodine was observed and 7 mass% iodine in the form of iodate was incorporated into the wasteform. These densified materials showed promising PCT performance (10^−5^–10^−7^ g∙m^−2^∙d^−1^).

A detailed mechanistic study showed that there are three main leach regimes to consider (Coulon et al., 2017). In unsaturated conditions, iodine leach rates are high (10^−2^ g∙m^−2^∙d^−1^ at 50°C), controlled by surface reactions and diffusion. In the second regime, when the concentrations of calcium and phosphate ions become saturated at the solubility limit of Ca_10_(PO_4_)_6_(OH)_2_, the rate of iodine release decreased. The third regime corresponds to a residual alteration taking place when the saturation of Ca and P is achieved. At 50°C, this leach rate was equal to 10^−4^ g∙m^−2^∙d^−1^ and was independent of the hydroxyapatite-CaI chemical composition. If a simulated clay-equilibrated groundwater, which is enriched in Ca, is used to simulate repository conditions, a further order of magnitude reduction in iodine leach rate was observed (Coulon et al., 2017). The authors noted that open porosity remained in the samples used to carry out the mechanistic investigation. This implies that with further optimization a reduction in leach rate could be achievable. To date, calcium phosphate apatites for iodine have been limited to the bench scale with little evidence for industrialization of this wasteform.

#### 3.5.5 Alumina based wasteforms

Alumina-based substrates for radionuclide capture have been under development for decades (e.g., Clariant AC-6120) with recent work showing promise for both aqueous capture and gas phase capture (Daryl Haefner, 2007; Wang and Chu, 2018; Zuo et al., 2020; Alsalbokh et al., 2021; Muhire et al., 2021). The investigation of AgI-containing alumina wasteforms has been well established with the use of HIP with these wasteforms being previously demonstrated (Tanabe et al., 2010). More recently, further work looking at the effect of pre-treatment of the material prior to HIP has been reported (Masuda et al., 2016). In the study, an alumina material containing 10 mass% silver was loaded with iodine and consolidated by HIP, resulting in an iodine loading of 10.2 mass%.

**FIGURE 9:**
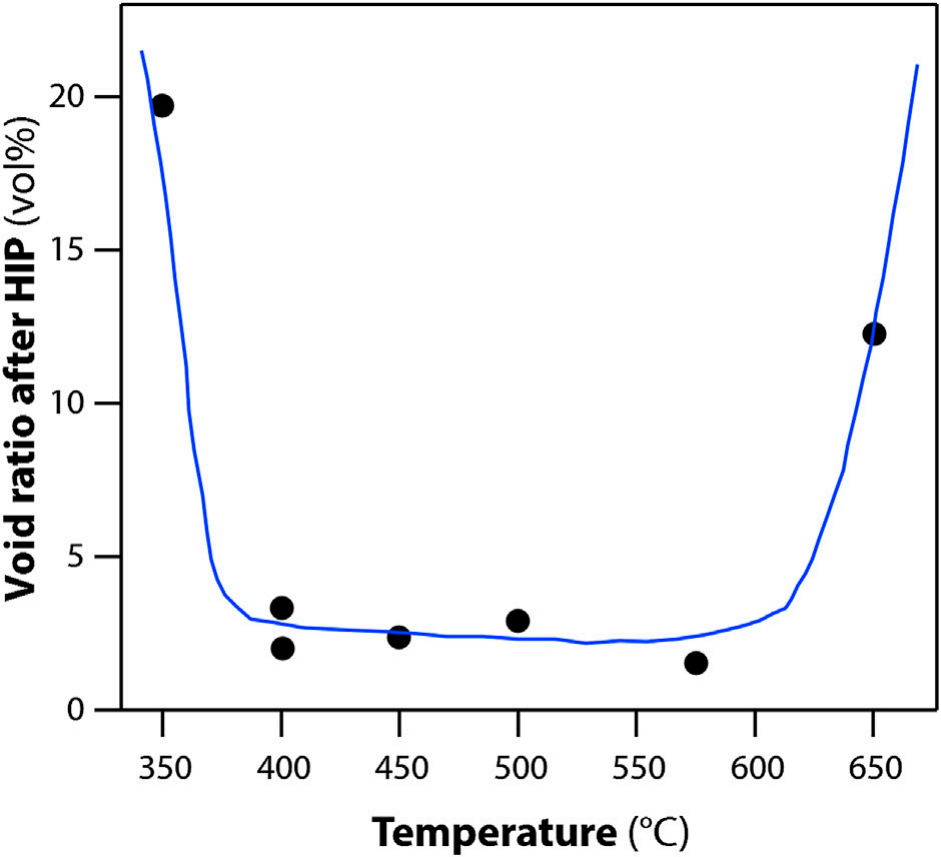
Effect of temperature during vacuum pretreatment on void ratio of solidified body. Pre-treatments: degassing under 5 × 10^–4^ Pa for 2 h at various temperatures; HIP treatments: 1200°C and 175 MPa for 3 h (Masuda et al., 2016).

Prior to HIP processing, a thermal treatment step was carried out. This involved heating the material up to 480°C for 6 h as well as a vacuum degassing to limit void space. The effect of varying the temperature on the void ratio after HIP is shown in Figure 9. In addition, a vacuum step was applied to remove any physiosorbed gases prior to sealing the can and HIPing. If this process was not carried out, then a void ratio of 15% was found in the consolidated HIP product. With the demonstration of HIP, alumina sorbents have a pathway to iodine wasteform production.

#### 3.5.6 Silica aerogel (AgS)

Aerogels provide a high specific surface area and ease of functionalization for iodine capture (Amonette and Matyáš, 2017). In the case of silver-functionalized silica-based aerogels (AgS), a stabilized AgI phase can be formed. The aerogel structure can then be directly densified through a collapse of the aerogel backbone. In recent years, HIP and HUP were used to condense AgS + I (Matyáš et al., 2016) along with durability studies (Asmussen et al., 2019). Whilst the HUP product was not fully densified, leaving open porosity of 16.9%, HIP resulted in densification of the silica-based wasteform to 3.3 × 10^3^ kg∙m^−3^ containing ∼22 mass% of iodine and no open porosity.

Durability tests on SPS-processed AgS + I wasteforms were investigated using the SPFT test and the addition of SiO_2_ as an additional binder led to improved durability compared with samples without SiO_2_ (Asmussen et al., 2019). Aerogel wasteforms densified *via* SPS have also shown improved iodine retention over their HIP counterparts; however, the two wasteforms have similar matrix dissolution rates (Reiser et al., 2022). While aerogels show promise, further maturation is required for production at scale. However, an aerogel capture material can utilize an industrially mature densification process.

#### 3.5.7 Perovskite

In recent years, iodine ceramics based on the perovskite structure have been explored in greater detail because of their potential high iodine incorporation and potential high durability. Perovskites that immobilize iodate have been reinvestigated in the context of iodine wasteforms, despite earlier reports of their synthesis in (Sleight and Ward, 1964). These wasteforms typically require iodate as their speciation and have the common form A_2_MIO_6_ (A = Ba, Sr, Ca; M = I, Na, K, Ag) (O’Sullivan et al., 2020). An incorporation of 25–40 mass% of iodine is achievable. Of note, the Ba_2_NaIO_6_ exhibited a high thermal stability, decomposing only above 1050°C, which is substantial for an iodine containing substance.

A defect perovskite structure A_2_BX_*x*_, Cs_2_SnI_6_ (66 mass%), has been reported (Scott et al., 2017). Consolidation by SPS occurred at a peak temperature of 250°C for 5 min. By thermogravimetric analysis the resulting material was stable up until 327°C. Mixed halide perovskites, Cs_2_SnI_*x*_Cl_5-*x*_ have also been reported (Zhu et al., 2018). As *x* increases, the thermal stability of the perovskite decreases from around 550°C to 300°C.

Silica has also been added to the consolidation of a defect perovskite Cs_3_Bi_2_I_9_ (K. Yang et al., 2021b). To form the Cs_3_Bi_2_I_9_, CsI and BiI_3_ were dissolved in gamma-butyrolactone, stirred for 6 h (H. Zhang et al., 2018a), and then crystalized using a solvent volatilized crystallization method (H. Zhang et al., 2017a). These were then consolidated by SPS, with a resulting perovskite loading between 30 and 70 mass%. Theoretical densities of 90.5–93.5% of the maximum were obtained.

Yang et al. showed that the core-shell approach can also be applied to perovskites (K. Yang et al., 2021b). Cs_3_Bi_2_I_9_ was included, up to 70 mass%, and mixed into a silica matrix to form GCM. A core-shell demonstration was carried out with a 20 mass% Cs_3_Bi_2_I_9_ encapsulated into amorphous silica through low-temperature SPS. Incongruent dissolution for Cs and I was observed in semi-dynamic leach testing along with a BiOI alteration layer that provided corrosion resistance. The core-shell sample had a 6× lower dissolution rate than the GCM.

**FIGURE 10:**
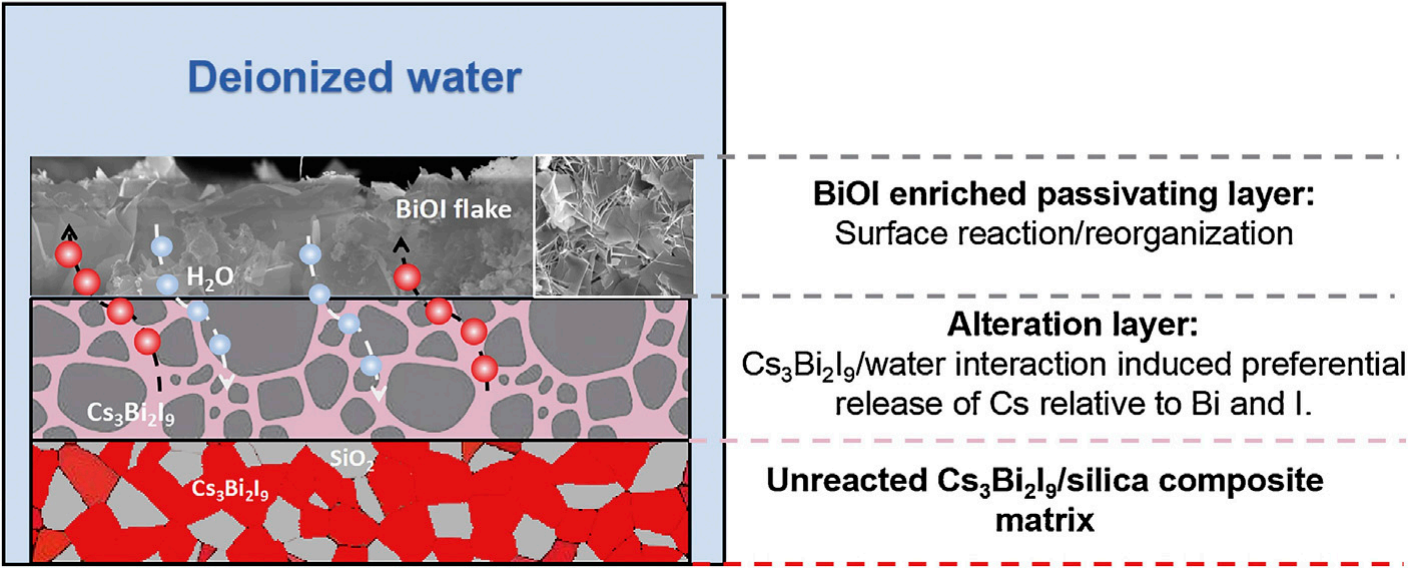
Schematic of durability mechanism for Cs_3_Bi_2_I_9_ silica composite (K. Yang et al., 2021b).

Durability studies in DI water showed that an alteration layer formed, which comprised of depleted Cs relative to Bi and I. In addition, an BiOI enriched passivating layer forms on the top of the alteration layer (Figure 10). The highest 70% loaded Cs_3_Bi_2_I_9_-silica composite showed the lowest leach rate, which was attributed to the observation of the protective BiOI layer. Studies of a CsPbI_3_ synthesized in solution then pelletized *via* SPS showed that Cs and I were leached at a faster rate than the Pb (Bryce et al., 2022). This incongruent dissolution was also observed in dissolution studies of a solution-prepared Cs_3_Bi_2_I_9_ phase that had been embedded into a hydroxyapatite matrix by spark plasma sintering. In both static and semi-dynamic leaching tests Cs and I were incongruently released in both the pure phase and from the hydroxyapatite composite. In this case the normalized Cs release rate was faster than that of I and Bi, due to the strength difference between Cs–I and Bi–I bonds as well as the formation of insoluble BiOI precipitates (Yang et al., 2022).

The recent work in this area has highlighted potentially promising materials from the perspective of durability. However, the starting materials required for the synthesis of perovskites involve adding steps to the processing of iodine-containing wastes. Similar to other wasteforms, investigations of how the leaching mechanism change in the presence of reducing agents and varying pH will be important to understand the long-term durability of these materials.

#### 3.5.8 Copper iodide (CuI)

Given that most promising capture methods result in the formation of AgI, the use of CuI as a wasteform would either necessitate a AgI–CuI conversion step or a change in philosophy for capture materials to directly capture as CuI from Cu-containing sorbents. The direct disposal of CuI as a iodine wasteform has been previously discussed (Burger et al., 1981; Taylor, 1990), but more recent reports have explored creating a densified CuI wasteform using industrially available techniques. Up to 83% of the theoretical density can be achieved using HIP (Vance et al., 2018). These materials are interesting because of the high iodine loading in the wasteform. Durability studies were carried out both in DI water and with the presence of 0.5 g of Cu, Ni, and Fe to simulate groundwaters. Under the reducing iron conditions, the release of iodide was substantial similar to iodine releases from wasteforms containing AgI.

### 3.6 Wasteform conversion summary

As evidenced by the extensive work in iodine wasteform development the waste immobilization community has shown ingenuity and diligence in converting the small number of well-developed iodine capture materials into wasteforms using a vast range of techniques. Specific selection of wasteforms will be highly dependent on disposal scenarios, but further development is anticipated that will include advances in materials science and maturation of conversion technologies.

## 4 Summary

This paper provided a comprehensive update on the rapidly evolving field of iodine wasteforms. The relative merits of various consolidation technique options have been considered and any report relating to iodine wasteforms has been captured. Other authors have made progress in connecting the most promising wasteforms and capture materials together to highlight some of the most fruitful potential options. Furthermore, durability reviews have provided a summary of leach data for various wasteforms, many of which include AgI or NaI as the immobilized form of iodine (Reiser et al., 2022). Despite several differences between the chemical durability experimental parameters, this repository of data provides a fairly comprehensive overview which indicate that several AgI-containing and NaI-containing wasteforms show low dissolution rates including AgI–Bi_2_O_3_–P_2_O_5_–ZnO glasses (<2.6 × 10^−4^ g∙m^−2^∙d^−1^) (Yang et al., 2015), SPS-processed and I-loaded Ag^0^-functionalized silica aerogels (1.2 × 10^−1^ to 1 × 10^0^ g∙m^−2^∙d^−1^) (Asmussen et al., 2019), HIPed I-loaded Ag-sodalites (4.3 × 10^−4^–4.1 × 10^ ^ g m^−2^∙d^−1^) (Maddrell et al., 2019), and HIPed I-loaded Ag-mordenites (4.3×10^0^ to 1.4 × 10^2^ g∙m^−2^∙d^−1^) (Yang et al., 2015).

Some of the key advances in recent years of the work reported include: exploring methods of reducing the vitrification temperature of glass-based wasteforms to avoid iodine volatilization but retain durability; simplify sodalite formation and consolidation without the formation of unwanted phases, furthering mechanistic understanding of apatite durability; and advancing development of highly durable, yet synthetically challenging, perovskite materials.

However, several gaps still exist and more work is needed in several instances such as: increasing mechanistic understanding of the leaching mechanisms as the materials have varied progresses; finding a clear link between capture material and wasteform for many of the wasteform concepts, especially those reliant on specific iodine speciation; and optimizing many wasteform concepts that have yet to be fully optimized with respect to density, therefore providing opportunity for increasing durability.

Industrially mature technologies are available for the conversion of capture materials to wasteforms such as HUP, HIP, and SPS. A primary challenge of the iodine wasteform community moving forward will be finding the balance between wasteform performance and feasibility of wasteform production at scale. Additionally, understanding the durability of the most promising wasteforms under realistic disposal conditions is a requirement for the field to take the next step towards credible wasteform options and enable optimization of consolidation conditions.
